# Preparation of *Astragalus membranaceus* Millet Rice Crust and Evaluation of Nutritional Components and Physicochemical Properties

**DOI:** 10.1155/ijfo/2594859

**Published:** 2025-12-12

**Authors:** Mengyu Cui, Yong Qin, Yufan Shang, Yakun Li, Huimin Ma, Jinghong Wang, Junling Zhu

**Affiliations:** ^1^ College of Food Science and Engineering, Shanxi Agricultural University, Taigu, Shanxi, China, sxau.edu.cn; ^2^ Shanxi Province Key Laboratory Cultivation Base of Minor Crops Nutrition and Healthy Food Development, Taigu, Shanxi, China

**Keywords:** *Astragalus membranaceus*, millet, nutritional evaluation, physicochemical properties, rice crust

## Abstract

*Astragalus membranaceus* is classified as a medicinal and food homologous substance, noted for its dense nutritional profile. Its main components include flavonoids, polysaccharides, and saponins. Millet, as a coarse cereal, is rich in protein, amino acids, and a variety of vitamins. We developed a nutritionally enhanced rice crust using *Astragalus membranaceus* and millet as the main raw materials. This study investigated the effect of *Astragalus membranaceus* addition on the nutritional composition and physicochemical properties of rice crust. Compared to the control millet rice crust (MRC) and a commercial rice crust (CRC), the millet rice crust with *Astragalus membranaceus* (AMRC) exhibited enhanced nutritional value, including higher protein (8.87 g/100 g DW) and ash (0.69 g/100 g DW) contents. Furthermore, its texture, color, and flavor were found to be acceptable. The morphological characteristics and functional groups of the rice crust samples were systematically analyzed using scanning electron microscopy (SEM) and Fourier transform infrared spectroscopy (FTIR); the results indicated that the AMRC sample possessed favorable structural characteristics. Analysis of bioactive compounds confirmed that they were present at higher concentrations in AMRC compared to the control. Based on free radical scavenging assays such as DPPH and ABTS, it was demonstrated that the AMRC exhibited significantly enhanced antioxidant activity. The study found the AMRC to be nutrient‐rich with health benefits, supporting its development as functional leisure food.

## 1. Introduction

Rice crust is a traditional snack enjoyed by consumers. While commercial varieties are primarily made from millet and rice, providing basic nutrients like lipids, proteins, and vitamins, they are generally low in unique functional components, resulting in limited nutritional value. With growing economic affluence, people′s lifestyle priorities have shifted, leading to a greater appreciation for the benefits of coarse cereals [1]. As a food crop, millet is gluten‐free, which leads to poor dough formability and limits its applications. This, in turn, hinders the development of coarse cereal baked goods due to their often inferior sensory quality. Therefore, research focused on millet‐based foods, particularly baked products, is essential to improve processability, enhance consumer acceptance, and foster the growth of the millet baking industry [2]. In recent years, there has been a growing trend of incorporating functional ingredients into baked goods. This trend is driven by a focus on enhancing nutritional value, with research analyzing their impact on the value‐added properties of the final products. Pawde et al. [3] formulated cellulose‐rich biscuits by incorporating pitaya powder and determined their cellulose and phenol content. Hidalgo et al. [4] investigated the effect of incorporating red beetroot pomace extract into the biscuit formulation on the total polyphenol content and antioxidant capacity. Indrianingsih et al. [5] investigated mangosteen peel extract for its effects on the antioxidant activity and antidiabetic potential of sorghum‐based biscuits.

The concept of “medicine and food homology” underscores the intrinsic connection between food and medicine. Substantial evidence indicates that items classified under this concept serve a dual purpose: they provide basic sustenance and fulfill healthcare and wellness preservation needs. Professor Bin Cong, an Academician of the Chinese Academy of Engineering, defined the concept of “homology of food and medicine” as referring to substances that integrate both pharmacological and nutritional functions. He further specified that the medicinal components within such substances should be safe for long‐term consumption without causing adverse effects. As a medicine–food homologous substance, *Astragalus membranaceus* contains functional components like flavonoids, polysaccharides, and saponins, which contribute to its various biological functions. These include health maintenance, disease prevention, and protective effects on multiple systems such as the cardiovascular and nervous systems [6–9]. Wei et al. [10] developed fortified biscuits using *Astragalus membranaceus* and cranberry powder as key ingredients and evaluated their antioxidant properties by measuring DPPH and hydroxyl radical scavenging activities. *Astragalus membranaceus* powder possesses significant nutritional value. Its application in food processing enhances the utilization of medicinal and edible homologous resources, thereby meeting the growing public demand for health‐oriented food products [11]. Song et al. [12] observed a significant increase in physiologically active compounds and a concomitant enhancement in antioxidant capacity by supplementing the preparation of germinated black rice porridge with *Astragalus membranaceus* extract and fermented *Astragalus membranaceus* extract. Wang et al. [13] employed various treatment methods to enhance the physicochemical properties and antioxidant activity of *Astragalus membranaceus* honey wine. The success of these methods demonstrates the promising development prospects of products utilizing *Astragalus membranaceus* as a primary raw material.

This investigation involved the preparation of millet rice crust with *Astragalus membranaceus* (AMRC) for comparison with millet rice crust (MRC) and commercially available rice crust (CRC). The basic nutritional composition of the three rice crusts was quantified. Furthermore, texture profile analysis (TPA) and color measurement were employed using a texture analyzer and a portable colorimeter, respectively. The taste profiles and volatile organic compounds of the three rice crusts were analyzed using an electronic tongue and an electronic nose system, respectively. The morphological characteristics and functional group compositions of the three rice crust powders (AMRC, MRC, and CRC) were characterized using scanning electron microscopy (SEM) and Fourier transform infrared spectroscopy (FTIR), respectively. In addition, the bioactive component content and antioxidant activity of the three rice crusts were determined.

## 2. Materials and Methods

### 2.1. Materials

*Astragalus membranaceus* was obtained from Hengmaoyuan Agricultural Development Co. Ltd. (Hunyuan County, Shanxi, China). Qinzhou Huang millet was purchased from Jinwei Food Co. Ltd. (Qinxian County, Shanxi, China). The commercial control sample, Qinzhou Huang MRC, was sourced from a local supermarket.

Common ingredients, including low‐gluten flour (Fujian Lingyuan Xinyi Food Co. Ltd., Batch No. 20250120A), salt (Shanxi Jinjiu Salt Co. Ltd., Batch No. 20241228G435), baking powder (Hubei Angel Yeast Co. Ltd., Batch No. 20240927Y32), and corn oil (Jiangxi Qinglong High‐Tech Oil Co. Ltd., Batch No. 20241229AL97), were also procured from a local supermarket.

### 2.2. Preparation of Rice Crusts

Based on the results of preliminary experiments, we established the following formulation: *Astragalus membranaceus* powder (8 g), millet flour (60 g), low‐gluten flour (40 g), baking powder (1.0 g), and salt (0.9 g).

**Figure 1:**
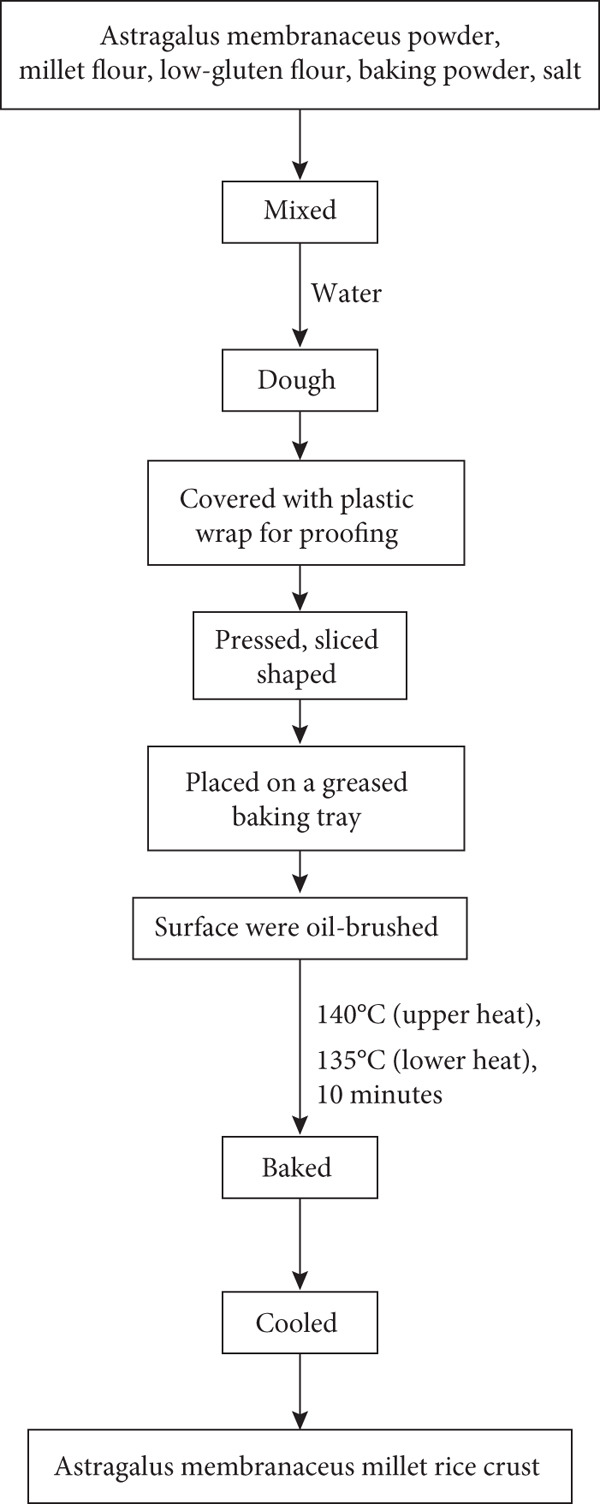
Flowchart diagram for the production of rice crust.

For the preparation process of AMRC, according to the formulated recipe, *Astragalus membranaceus* powder, millet flour, and low‐gluten flour were accurately weighed and thoroughly mixed. Baking powder, salt, and water were then added to form a dough. The dough was covered with plastic film and allowed to rest for 10 min. Subsequently, it was pressed, sliced, and shaped before being transferred to a greased baking tray. The surface was brushed with oil, and the samples were baked in a preheated oven at 140°C (upper heat) and 135°C (lower heat) for 10 min. The experimental workflow is depicted schematically in Figure 1.

The control group, designated as MRC, was prepared by omitting *Astragalus membranaceus* powder from the standard formulation while keeping all other steps unchanged. CRC was also included as an additional control for comparison.

### 2.3. Determination of Basic Nutritional Indexes

The basic nutritional composition was analyzed according to the official AOAC methods [14]. The moisture content was measured by employing the direct drying method. The content of basic nutrients was expressed in grams per 100 g of dry rice crust (g/100 g). The ash content was determined by the direct ashing method. Protein content was determined using the Kjeldahl method with a conversion factor of 6.25 [15]. The total fat content (including both free and bound fat) was determined by acid hydrolysis. The carbohydrate content was determined by the subtraction method, which involves subtracting the contents of protein, fat, ash, and moisture from the total weight. After being measured in triplicate, all basic nutritional indicators were expressed as mean values for analysis.

### 2.4. Texture Analysis

The TPA was performed using a texture analyzer (TA.New plus, ISENSO, United States) according to the method of Arepally et al. [16], with minor modifications. TPA was performed using a T42 probe with the following parameters: a pretest speed of 2 mm/s, a test speed of 60 mm/min, a posttest speed of 2 mm/s, a trigger force of 0.4 N, and a compression level of 90%. The texture properties of the rice crust, including hardness, cohesiveness, springiness, and chewiness, were analyzed. All measurements were performed in triplicate, and the results were expressed as mean values.

### 2.5. Color Analysis

A portable colorimeter (WSC‐3B, Shanghai Wuguang/Yidian, China) was used. Following calibration, the *L*, *a*, and *b*∗ values were measured by placing the probe on the sample. Each sample was measured three times, and the results were averaged.

### 2.6. Taste Analysis

The analysis was conducted following the method of Lu et al. [17] with slight modifications. Briefly, 2 g of the sample was homogenized with 100 mL of distilled water and then centrifuged at 10,000 r/min for 10 min. The resulting supernatant was transferred to the specialized sample cup of an electronic tongue (SA402B, Insent, Japan) for measurement. Each sample was analyzed in triplicate, and the average value was calculated.

### 2.7. Odor Analysis

The volatile profiles of the three rice crust samples were analyzed using an electronic nose (PEN 3, AIRSENSE, Germany) according to a slightly modified method from Zhao et al. [18]. Briefly, 5 g of each sample was sealed in a 50‐mL centrifuge tube and equilibrated at room temperature for 30 min before headspace sampling.

The electronic nose was operated under the following conditions: the sampling interval was set to 1 s/group, the sensor self‐cleaning time was 80 s, the sample injection time was 5 s, the carrier gas flow rate was 400 mL/min, and the analysis sampling time was 80 s. Each sample was measured in triplicate, and the average value was used for analysis.

### 2.8. SEM Analysis

The sample powder was evenly dispersed on conductive adhesive mounted on a sample stub. Subsequently, the sample was sputter‐coated with a layer of gold in a vacuum evaporator and then observed under a scanning electron microscope (SEM, Hitachi SU‐3500, Japan) at an appropriate accelerating voltage to analyze its morphology.

### 2.9. FTIR Analysis

FTIR (IRXross, Shimadzu, Japan) was performed to identify the functional groups in the rice crust samples over the wavenumber range of 4000–400 cm^−1^.

### 2.10. Determination of Bioactive Components

#### 2.10.1. Determination of Total Flavonoids

The sample solution was prepared according to a method adapted from Cui et al. [19]. Briefly, 3 g of the sample was extracted with 80% ethanol at a solid‐to‐liquid ratio of 1:25 (g:mL) under ultrasonication at 45°C (100 W) for 100 min. The resulting mixture was filtered and centrifuged at 3000 r/min for 10 min. A 5‐mL aliquot of the supernatant was taken for subsequent analysis.

The total flavonoid content (TFC) was determined according to the method of Ji et al. [20] with slight modifications. The absorbance was measured at 510 nm, a standard curve was prepared using rutin, and the results were expressed as milligrams of rutin equivalent per gram of dry weight (mg RE/g DW). All measurements were performed in triplicate, and the mean values were reported.

#### 2.10.2. Determination of Total Polysaccharides

The sample solution was prepared following the method of Li et al. [21] with minor modifications. Briefly, 10 g of the sample was extracted with distilled water (1:10, g:mL) at 85°C for 2 h. After centrifugation at 4500 r/min for 10 min, the supernatant was collected and mixed with two volumes of 95% ethanol. The solution was shaken thoroughly and stored at 4°C overnight. The mixture was then centrifuged at 5000 r/min for 10 min. The resulting precipitate was redissolved in distilled water and diluted to a final volume of 100 mL. A 1‐mL aliquot of this solution was used for analysis.

The total polysaccharide content was determined using a method adapted from Chen et al. [22]. A standard curve was prepared with glucose, and the results were expressed as milligrams of glucose equivalents per gram of dry weight (mg GE/g DW). All measurements were performed in triplicate, and the results were expressed as the mean values.

#### 2.10.3. Determination of Total Saponins

The sample solution was prepared using a method adapted from Hu et al. [23]. Briefly, 10 g of the sample was extracted with 80% ethanol (1:10, g:mL) at 50°C for 1.5 h. The mixture was then filtered and centrifuged at 3500 r/min for 20 min. A 0.2‐mL aliquot of the resulting supernatant was taken for analysis.

The total saponin content was determined according to a method adapted from de Aguiar et al. [24] with minor modifications. A standard curve was prepared using *Astragalus membranaceus* IV, and the results were expressed as milligrams of *Astragalus membranaceus* IV equivalent per gram of dry weight (mg/g DW). All measurements were performed in triplicate, and the results were expressed as the mean values.

### 2.11. Antioxidant Determination

The sample solution was prepared based on the method of Gramza‐Michałowska et al. [25] with minor modifications. Briefly, 1 g of finely ground rice crust was homogenized in 10 mL of an 80% methanol solution. The mixture was then subjected to ultrasonic‐assisted extraction (40 kHz) for 20 min, followed by centrifugation at 3000 r/min for 15 min. The resulting supernatant was filtered and diluted to a final volume of 100 mL to obtain the test solution.

The DPPH free radical scavenging activity was determined following the method of Szydłowska‐Czerniak et al. [26] with slight modifications. Briefly, a 0.2 mmol/L DPPH solution was prepared in anhydrous ethanol. Then, 2 mL of this solution was mixed with 2 mL of the sample extract. After incubation in the dark for 30 min, the absorbance was measured at 517 nm. A control group used anhydrous ethanol instead of the DPPH solution, and a blank group used methanol instead of the sample solution. All measurements were performed in triplicate, and the scavenging rate was calculated using Formula (1):

(1) DPPH free radical scavenging rate %=1−A1−A2A0×100%

where *A*_1_ is the average absorbance of the sample, *A*_2_ is the average absorbance of the control group (anhydrous ethanol instead of DPPH solution), and *A*_0_ is the average absorbance of the blank group (methanol instead of sample solution).

The ABTS radical scavenging capacity was determined based on the method of Re et al. [27] with slight modifications. Briefly, the stock solution was prepared by mixing equal volumes of 7 mmol/L ABTS solution and 2.45 mmol/L potassium persulfate (K_2_S_2_O_8_) solution, followed by incubation in the dark at room temperature for 16 h. Prior to use, the stock solution was diluted with 0.01 mol/L phosphate buffer (pH 7.4) to an absorbance of 0.72–1.20 at 734 nm to obtain the working solution. Subsequently, 1 mL of the sample extract was mixed with 4 mL of the ABTS working solution. After reacting for 6 min, the absorbance was measured at 734 nm. A control (phosphate buffer instead of ABTS) and a blank (methanol instead of sample) were included. Measurements were performed in triplicate, and the scavenging rate was calculated using Formula (2):

(2) ABTS free radical scavenging rate %=1−A1−A2A0×100%

where *A*_1_ is the average absorbance of the sample, *A*_2_ is the average absorbance of the control group (phosphate buffer instead of ABTS solution), and *A*_0_ is the average absorbance of the blank group (methanol instead of sample solution).

The hydroxyl radical scavenging activity was determined using a modified version of the method described by Ding et al. [28]. Briefly, 2 mL of 9 mmol/L ferrous sulfate was mixed with 2 mL of 9 mmol/L salicylic acid–ethanol solution and 2 mL of the test sample in a test tube. After vortexing, 2 mL of 20 mmol/L hydrogen peroxide was added to initiate the reaction. The mixture was then incubated in a water bath at 37°C for 30 min, and the absorbance was measured at 510 nm using pure water as a blank. All assays were performed in triplicate, and the scavenging rate was calculated using Formula (3):

(3) X=1−x1−x2x0×100%

where *X* is the hydroxyl radical scavenging rate (%), *x*_1_ is the mean absorbance of the sample, *x*_2_ is the mean absorbance of the control (pure water replacing H_2_O_2_), and *x*_0_ is the mean absorbance of the blank control (pure water).

The superoxide anion radical (O_2_^−^) scavenging activity was determined based on the pyrogallol autoxidation method described by Papadaki et al. [29] with slight modifications. Briefly, 2.5 mL of the sample solution was mixed with 4 mL of 0.05 mol/L Tris‐HCl buffer (pH 8.2) and equilibrated in a 25°C water bath for 20 min. Then, 1 mL of 25 mmol/L pyrogallol solution was added to initiate the reaction (using double‐distilled water instead of the sample for the blank control). After vortexing, the reaction proceeded at 25°C for 5 min and was terminated by adding 0.1 mL of 8 mol/L HCl. The absorbance was measured at 320 nm. All assays were performed in triplicate, and the scavenging rate was calculated using Formula (4):

(4) Superoxide anion radical scavenging rate %=A0−A1A0×100%

where *A*_0_ is the average absorbance value of the blank control and *A*_1_ is the average absorbance value of the sample.

### 2.12. Statistical Analysis

All treatments were conducted in triplicate, and data were presented as mean ± standard deviation. Statistical significance was determined by one‐way analysis of variance (ANOVA) followed by Duncan′s multiple comparison test using IBM SPSS Statistics 27.0. A value of *p* < 0.05 was considered statistically significant. Origin 2024 software was used for drawing.

## 3. Results and Discussion

### 3.1. Basic Nutritional Indexes

The nutritional compositions of the three rice crusts are presented in Table 1. The results indicated that the moisture content of AMRC was higher than that of MRC but lower than that of CRC. This variation in moisture content among the products could be attributed to differences in both the baking process and the initial water content of the raw materials [30]. The ash, protein, and carbohydrate contents in AMRC were significantly higher than those in the other two rice crusts. These results suggested that the incorporation of *Astragalus membranaceus* effectively enhanced the mineral and protein content of the rice crust [10]. The carbohydrate content in AMRC was higher than in the other two rice crusts. This elevated level could be attributed to the calculation method, as carbohydrates were determined by difference (i.e., 100% minus the percentages of moisture, ash, protein, and fat). This approach included dietary fiber within the carbohydrate value. Furthermore, substituting wheat flour with millet flour inherently increased the dietary fiber content, which consequently contributed to a higher calculated carbohydrate value for AMRC [31]. Furthermore, the presence of native polysaccharides in *Astragalus membranaceus* was also a contributing factor to the observed results [32]. Furthermore, the baking process used in this experiment reduced the oil content of the final product. Consequently, the fat content in the AMRC was lower than in the CRC. This reduction in fat likely resulted in a higher relative proportion of carbohydrates in the AMRC on a dry weight basis. Quantification of the basic nutritional components in the three rice crust varieties revealed that the sample supplemented with *Astragalus membranaceus* powder possessed a superior nutritional profile, suggesting its potential to contribute to a balanced diet.

**Table 1:** Determination results of basic nutritional indexes.

**Sample**	**Moisture (g/100 g)**	**Ash (g/100 g DW)**	**Protein (g/100 g DW)**	**Fat (g/100 g DW)**	**Carbohydrate (g/100 g DW)**
AMRC	3.52 ± 0.04^b^	0.69 ± 0.02^a^	8.87 ± 0.15^a^	19.73 ± 0.15^c^	67.19 ± 0.04^a^
MRC	3.38 ± 0.07^c^	0.63 ± 0.02^b^	8.50 ± 0.10^b^	20.56 ± 0.31^b^	66.93 ± 0.28^a^
CRC	3.70 ± 0.04^a^	0.57 ± 0.02^c^	8.17 ± 0.06^c^	24.83 ± 0.40^a^	62.73 ± 0.40^b^

*Note:* After the value, different letters indicate significant difference (*p* < 0.05).

### 3.2. Texture Analysis

TPA is an objective method used to quantify the physical properties of food, such as hardness, cohesiveness, springiness, and chewiness. The results provide crucial insights into the sensory quality and overall acceptability of the rice crust [33]. Hardness is defined as the force required to achieve a given deformation of the rice crust sample. As a key textural attribute, it directly influences the consumer′s perception of product quality, freshness, and overall satisfaction [34]. Cohesiveness quantifies the strength of the internal structural bonds within the rice crust sample, indicating how well it withstands deformation before breaking. Springiness measures the degree to which the sample recovers its original height and shape after the deforming force is removed during compression [35]. Chewiness is defined as the energy required to masticate a solid food to a state ready for swallowing [36].

As shown in Table 2, the AMRC sample exhibited significantly higher hardness and chewiness but lower cohesiveness and springiness than both the MRC and CRC samples. It was hypothesized that the observed dough stiffening resulted from water absorption by the trace starch in the *Astragalus membranaceus* powder. This absorption facilitated the formation of a gluten‐analogous network structure, thereby increasing the chewiness of the product [37]. Furthermore, the observed increase in the hardness of the rice crust can be attributed primarily to the high dietary fiber content in *Astragalus membranaceus* [38]. The observed texture of the CRC was attributed to its high fat content, which had a tenderizing effect. This was because the fat coated the gluten proteins, which interfered with gluten development by effectively shortening the gluten network strands and limiting the formation of a strong, continuous structure [39]. The AMRC sample exhibited significant differences (*p* < 0.05) from both the MRC and CRC samples for all tested texture parameters, including hardness, cohesiveness, springiness, and chewiness.

**Table 2:** Determination results of texture characteristics.

**Sample**	**Hardness (N)**	**Cohesion (ratio)**	**Springiness (mm)**	**Chewiness (mJ)**
AMRC	12.77 ± 0.17^a^	0.03 ± 0.01^c^	0.08 ± 0.01^c^	0.38 ± 0.07^a^
MRC	9.88 ± 0.51^b^	0.06 ± 0.02^b^	0.17 ± 0.05^b^	0.20 ± 0.03^b^
CRC	6.42 ± 0.21^c^	0.10 ± 0.02^a^	0.24 ± 0.02^a^	0.13 ± 0.02^b^

*Note:* After the value, different letters indicate significant difference (*p* < 0.05).

### 3.3. Color Analysis

The color parameters of the three samples are presented in Table 3. The AMRC exhibited a significantly higher *L*∗ value (lightness) than the CRC (*p* < 0.05), but not than the MRC, indicating it had the brightest appearance. This lower lightness in CRC was most likely due to more extensive Maillard reaction products, such as melanoidins, formed due to its higher processing temperature [40]. The CRC sample exhibited a significantly higher *a*∗ value (redness/greenness) than the other two varieties (*p* < 0.05), indicating a deeper red color compared to the self‐made samples. This was hypothesized to be due to the formation of colored reaction products in CRC, such as red amine–quinone adducts derived from polyphenol–protein interactions [41]. The AMRC sample exhibited a significantly higher *b*∗ value (yellowness/blueness) than the MRC and CRC samples (*p* < 0.05). This enhanced yellowness was likely due to the presence of flavonoids in *Astragalus membranaceus*, such as calycosin, which contribute to the product′s color [42]. The color differences among the three rice crust samples could also be attributed to the specific type of oil used in their respective formulations. Variation in oil type influenced the final product′s color, consequently altering its lightness (*L*), redness (*a*), yellowness (*b*), and overall color profile [43].

**Table 3:** Color determination result.

**Chromatic value**	**AMRC**	**MRC**	**CRC**
*L* ^∗^	66.7 ± 0.90^a^	66.28 ± 0.63^a^	62.23 ± 0.50^b^
*a* ^∗^	9.82 ± 0.21^b^	9.77 ± 0.21^b^	13.83 ± 0.48^a^
*b* ^∗^	41.92 ± 0.92^a^	40.04 ± 0.56^b^	39.62 ± 0.89^b^

*Note:* After the value, different letters indicate significant difference (*p* < 0.05).

### 3.4. Taste Analysis

The electronic tongue mimics human taste perception by using multichannel taste sensors. The system employed in this study was composed of five specific sensors (C00, AE1, CA0, CT0, and AAE), each sensitive to different taste attributes. Their response profiles to the three rice crust samples were as follows: C00 for bitterness and bitter aftertaste, AE1 for astringency and astringent aftertaste, CA0 for sourness, CT0 for saltiness, and AAE for umami and richness [44]. Therefore, an electronic tongue was employed to evaluate the taste profiles and discriminate between the different rice crust samples.

**Figure 2:**
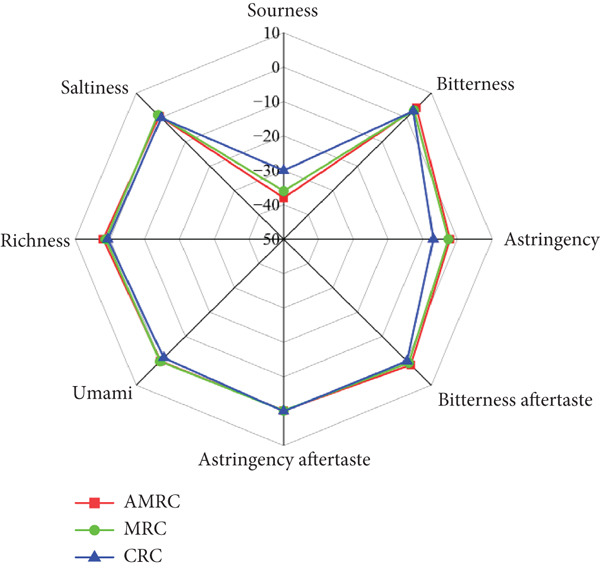
Comparison of the taste of different rice crusts.

**Figure 3:**
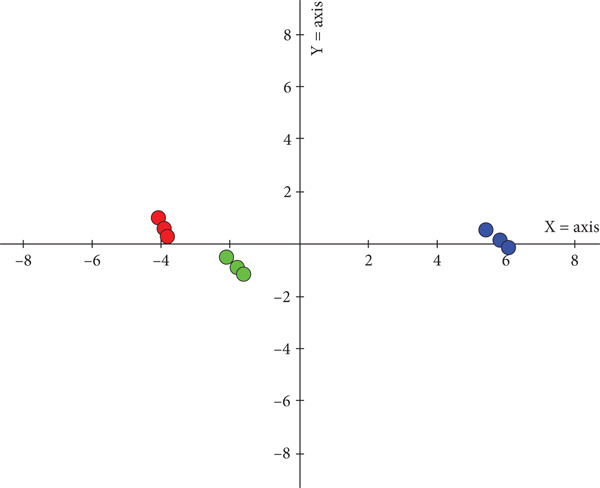
PCA of different rice crust tastes.

The taste analysis results presented in Figure 2 revealed significant differences in sourness and astringency among the three rice crusts. Specifically, the AMRC sample exhibited significantly lower sourness compared to the CRC (*p* < 0.05). Regarding astringency, AMRC′s value was significantly higher than that of CRC (*p* < 0.05) but was not significantly different from MRC (*p* > 0.05). This suggests that the addition of *Astragalus membranaceus* powder did not significantly alter the astringency relative to the base MRC. It was speculated that the higher astringency could be attributed to the polyphenols present in the millet flour itself, which are a primary source of this taste sensation [45, 46]. However, numerous studies have demonstrated that polyphenols in millet flour confer various health benefits, including potent antioxidant activity, improvements in blood lipid and glucose levels, promotion of intestinal health, and potential anticancer properties [47]. No significant differences (*p* > 0.05) were observed in saltiness, umami, bitterness, or richness among the three varieties. This similarity in key flavor profiles between the AMRC and commercial samples suggests that the AMRC likely maintained a high level of consumer acceptability. Principal component analysis (PCA) (Figure 3) showed clear discrimination among the three rice crust varieties, with distinct clusters confirming significant taste differences. This effective separation allowed for reliable flavor classification.

### 3.5. Odor Analysis

The electronic nose is a powerful tool for aroma profiling because it provides a holistic view of the volatile compounds, capturing the overall aroma profile and complex interactions within the sample [48]. The electronic nose utilized 10 sensors, each targeting specific volatiles: W1C (aromatics), W5S (nitrogen oxides), W3C (ammonia, aromatics), W6S (hydrides), W5C (olefins, aromatics, polar molecules), W1S (short‐chain alkanes), W1W (sulfur compounds), W2S (alcohols, aromatics), W2W (organic sulfides, aromatics), and W3S (long‐chain alkanes, aliphatics) [18].

**Figure 4:**
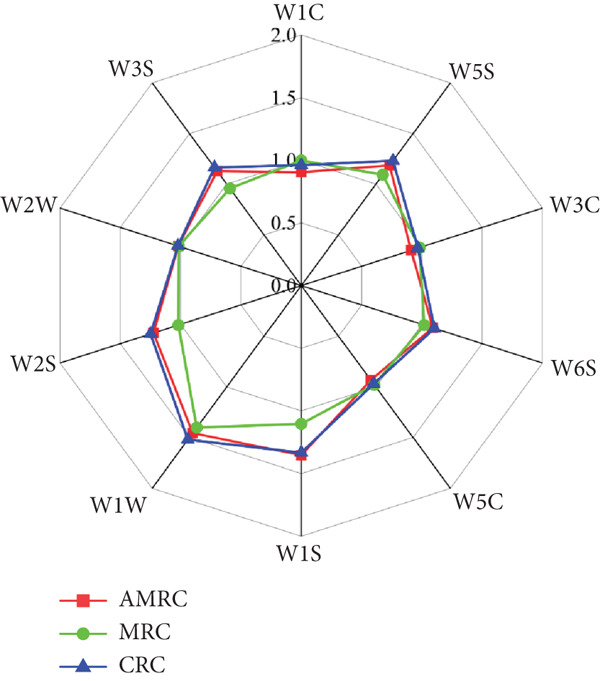
Electronic nose radar diagram of volatile components in different rice crusts. W1C, aromatics; W5S, nitrogen oxides; W3C, ammonia, aromatics; W6S, hydrides; W5C, olefins, aromatics, polar molecules; W1S, short‐chain alkanes; W1W, sulfur compounds; W2S, alcohols, aromatics; W2W, organic sulfides, aromatics; W3S,long‐chain alkanes, aliphatics.

As shown in Figure 4, the trend in signal changes from the 10 electronic nose sensors for AMRC and CRC corresponded to differences in volatile compound intensity. MRC′s volatile content was significantly lower than AMRC and CRC. The electronic nose radar chart revealed that the sensor responses for W1S (short‐chain alkanes), W2S (alcohols, aromatics), W3S (long‐chain alkanes, aliphatics), and W5S (nitrogen oxides) were higher in AMRC than in MRC. This suggests that the addition of *Astragalus membranaceus* powder effectively increased the content of specific volatile compounds compared to the control sample without it [49]. In the odor analysis of *Astragalus membranaceus* cranberry biscuits conducted by Wei et al. [10], the signals of the W2W, W1C, W5S, W1S, and W1W sensors were enhanced compared to the control biscuits. These sensors correspond to organic sulfides, aromatic compounds, nitrogen oxides, short‐chain alkanes (e.g., methane), and inorganic sulfides, respectively. This enhancement was attributed to the addition of *Astragalus membranaceus* and cranberry.

**Figure 5:**
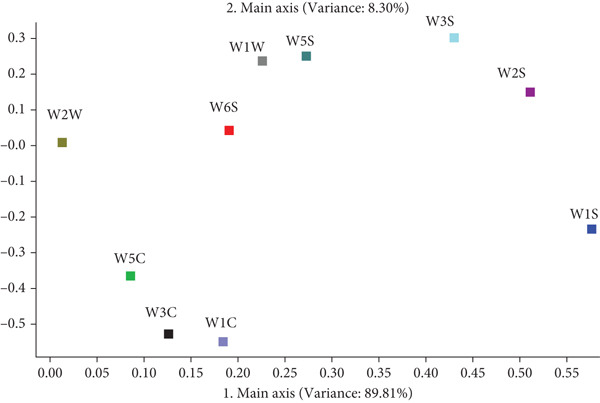
Contribution rate of principal component analysis of different rice crust flavor substances.

PCA contribution analysis (Figure 5) identified W1S short‐chain alkanes as the primary contributor to Principal Component 1, with W3S long‐chain alkanes and aliphatic compounds being the most influential for Principal Component 2.

**Figure 6:**
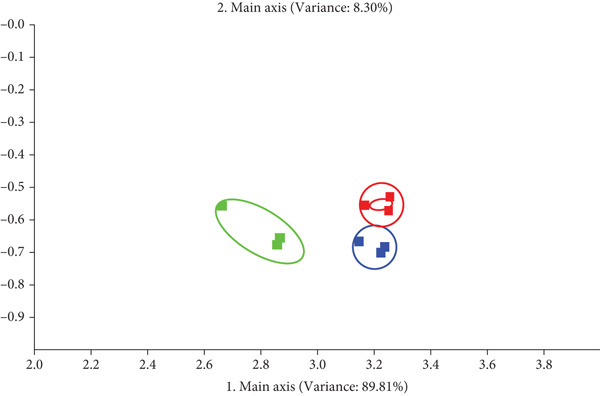
Two‐dimensional PCA images of electronic noses of different rice crusts. Red icon, AMRC; green icon, MRC; blue icon, CRC.

PCA results (Figure 6) demonstrated effective discrimination among the three rice crust types, indicating distinct profiles of volatile compounds. Principal Component 1 and Principal Component 2 together accounted for over 98% of the total variance, effectively capturing the major flavor differences among the samples.

### 3.6. SEM Analysis

**Figure 7:**
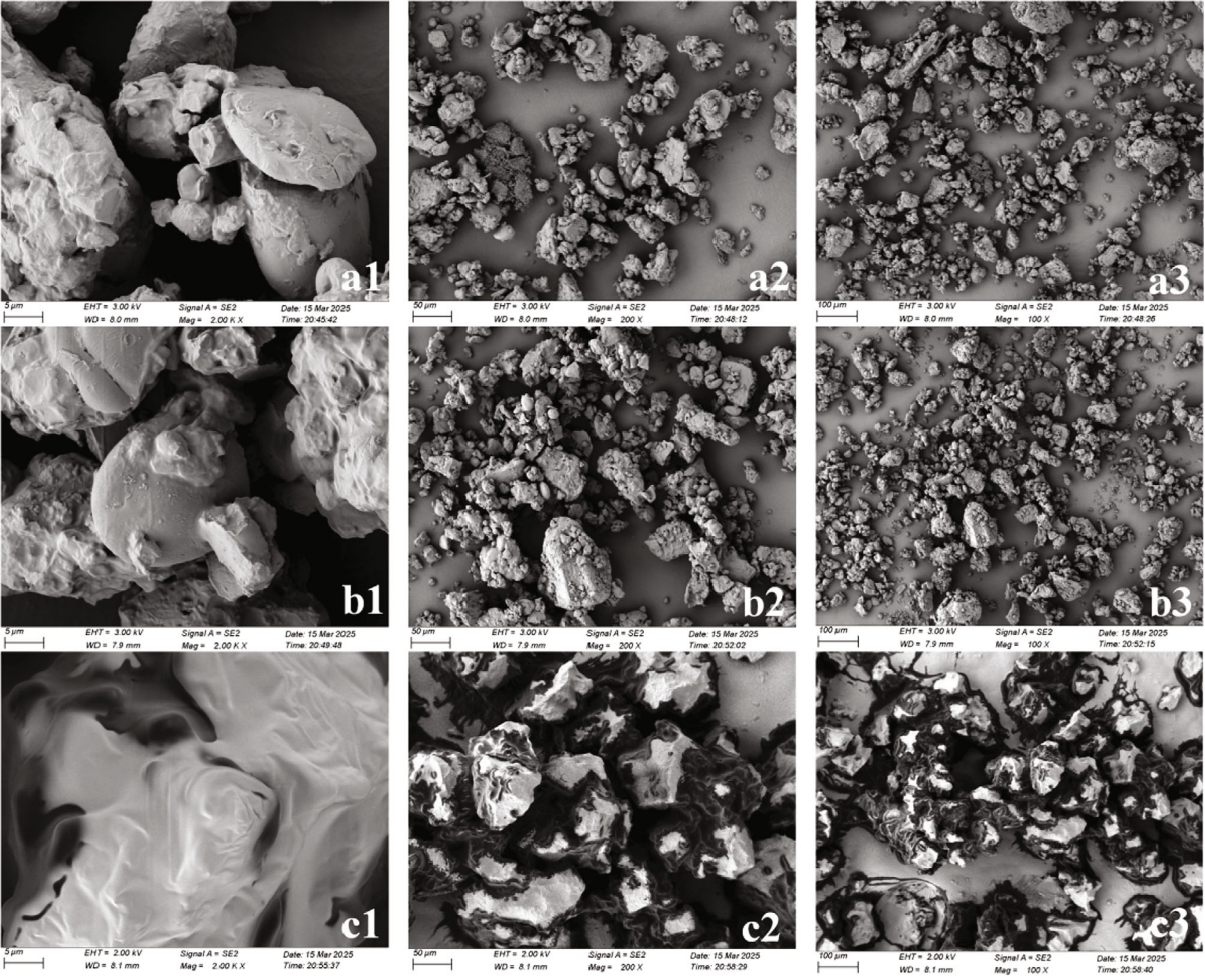
SEM of different sample powders. (a1–a3) AMRC; (b1–b3) MRC; (c1–c3) CRC.

The morphological features of the powdered samples were analyzed by SEM at different magnification levels (5, 50, and 100‐*μ*m scale bars). Images a1, a2, and a3 correspond to the AMRC sample at these respective scales; series b1–b3 and c1–c3 show the MRC and CRC samples under the same conditions. Dark, encapsulated substances were observed within the CRC sample in images c1–c3 of Figure 7. Given the previously determined high oil content of CRC, it was hypothesized that these dark areas represent oil enveloping the sample particles [50, 51]. SEM observations revealed that AMRC and MRC had similar morphological features, whereas CRC exhibited distinctly different structures. Notably, the AMRC particles possessed a more porous architecture. Two plausible explanations were proposed for this: first, the dietary fiber and other components in the *Astragalus membranaceus* powder increased the overall fiber content, promoting porosity; second, the *Astragalus membranaceus* powder and the millet flour may have undergone different reactions during the baking process [52]. Compared to AMRC and MRC, the CRC sample exhibited a higher oil content. The adhesive properties of the oil promoted stronger cohesion between particles, leading to more visible aggregation [53].

### 3.7. FTIR Analysis

FTIR was used to identify the functional groups present in the three rice crust samples and to explore the relationship between these functional groups and their nutritional properties. Carbohydrates (including fiber and sugars), lipids, and proteins are key parameters for evaluating rice crust quality. As major constituents, they play critical roles in determining both the sensory attributes and nutritional value. Carbohydrates provide energy and sweetness and influence the browning process during baking. Proteins enhance nutritional value and contribute to the product′s structure and texture. Lipids affect sensory characteristics and shelf life [54].

**Figure 8:**
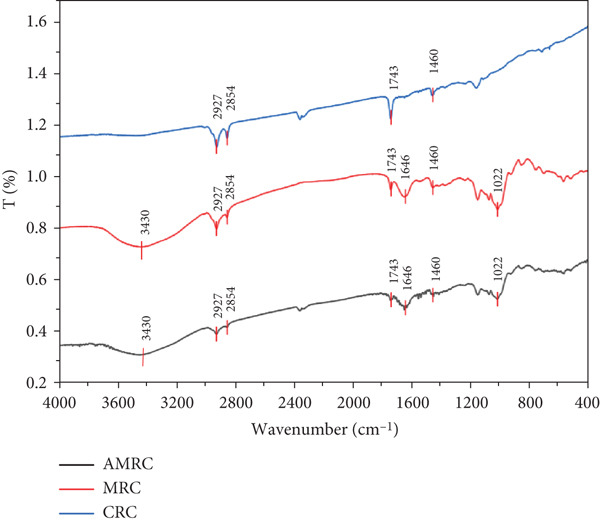
Infrared spectra of different rice crusts.

The FTIR spectra of the three rice crust samples are presented in Figure 8. The absorption peak observed at 3430 cm^−1^ was primarily attributed to the O‐H stretching vibration, characteristic of carbohydrates and other hydroxyl‐containing compounds. The absorptions in the range of 2854–2927 cm^−1^ were associated with C‐H stretching vibrations [30]. The characteristic absorption at 1743 cm^−1^ was assigned to the C=O stretching vibration of carbonyl groups; it was speculated that it may come from lipids [55, 56]. The characteristic absorption band at 1646 cm^−1^ was assigned to C=C stretching vibrations, indicative of the random coil conformation in protein [30, 56]. The absorption at 1460 cm^−1^ was characteristic of the −CH_2_ bending vibration. Furthermore, the peak observed at 1022 cm^−1^ was indicative of the starch conformation, which was influenced by both its ordered and amorphous structures [57, 58].

Furthermore, FTIR can help elucidate the antioxidant properties of the rice crusts by identifying key functional groups and linking them to known antioxidant mechanisms. For instance, hydroxyl groups (−OH) can donate hydrogen atoms to neutralize free radicals, thereby enhancing antioxidant activity. Similarly, the detection of carbonyl groups (C=O) is a hallmark of flavonoids, which are well‐known antioxidant compounds [59]. The bending vibration observed at 1460 cm^−1^ was characteristic of −CH_2_ groups, which may be associated with polysaccharide components known to contribute to antioxidant activity [60].

In this experiment, the FTIR spectra of AMRC and MRC showed nearly identical profiles, both distinctly different from CRC, though sharing similar functional groups. These results demonstrated that the three rice crust varieties shared similar chemical compositions.

### 3.8. Analysis of Bioactive Components

As shown in Table 4, the analysis of bioactive components revealed that the TFC was highest in AMRC, followed by CRC, and lowest in MRC. A one‐way ANOVA revealed significant differences in TFC among the three samples (*p* < 0.05). This result was attributed to the presence of characteristic flavonoids in *Astragalus membranaceus*, such as calycosin and formononetin [61]. Millet flour contains significant amounts of anthocyanins, flavonols, and other bioactive compounds [62]. The higher TFC in the CRC compared to the MRC was likely due to its composite formulation. The addition of various spice components in CRC probably contributed additional flavonoids [63]. Regarding the total polysaccharide content among the bioactive components, AMRC showed a higher level than both MRC and CRC. This increase was attributed to the inherent richness of polysaccharides in *Astragalus membranaceus* itself, as the addition of its powder directly enriched the final product′s polysaccharide content [64]. The total saponin content in AMRC was also significantly higher than that in the other two control samples (*p* < 0.05). This difference was presumably due to the inherent saponins in *Astragalus membranaceus* powder, which directly contributed to the elevated level in the final product [65]. Additionally, millet flour contained a small amount of saponins to increase the content of total saponins in AMRC [66].

**Table 4:** Determination results of bioactive components.

**Sample**	**Total flavonoids (mg/g DW)**	**Total polysaccharides (mg/g DW)**	**Total saponins (mg/g DW)**
AMRC	0.33 ± 0.01^a^	2.29 ± 0.08^a^	1.03 ± 0.02^a^
MRC	0.17 ± 0.04^c^	2.07 ± 0.07^a^	0.80 ± 0.02^b^
CRC	0.24 ± 0.02^b^	1.82 ± 0.17^b^	0.63 ± 0.01^c^

*Note:* After the value, different letters indicate significant difference (*p* < 0.05).

### 3.9. Antioxidant Analysis

The determination of the antioxidant capacity of foods has garnered growing interest, as it provides valuable insights into their antioxidant properties and allows for the quantification of antioxidant contributions [67]. DPPH and hydroxyl free radicals are among the most commonly employed indicators for assessing the efficacy of free radical scavenging in the food industry [10]. The data presented in Table 5 revealed that the AMRC sample possessed significantly superior antioxidant activity compared to both the MRC and CRC samples (*p* < 0.05). The significantly improved free radical scavenging capacity of the rice crust with *Astragalus membranaceus* powder could be explained by the potent antioxidant activity of its constituent flavonoids and polysaccharides, as established by previous studies on ABTS and DPPH radical scavenging [68, 69]. Similarly, Samuel et al. [70] demonstrated the antioxidant activity of *Astragalus membranaceus* extract through free radical scavenging assays, including ABTS and DPPH. Sheng et al. [68] isolated flavonoids from various sources of *Astragalus membranaceus* and evaluated their in vitro antioxidant capacity using DPPH and other free radical scavenging assays. The results confirmed that flavonoids from different *Astragalus membranaceus* varieties exhibited significant radical scavenging abilities.

**Table 5:** Determination results of antioxidant activity.

**Sample**	**DPPH scavenging rate (%)**	**ABTS** ^**+**^ **scavenging rate (%)**	**OH scavenging rate (%)**	**O**_**2**_^**−**^ **·scavenging rate (%)**
AMRC	76.84 ± 0.51^a^	75.9 ± 0.12^a^	16.77 ± 0.77^a^	53.52 ± 0.37^a^
MRC	47.31 ± 0.61^c^	50.14 ± 0.18^c^	13.11 ± 0.67^b^	45.65 ± 0.34^c^
CRC	66.26 ± 0.62^b^	69.89 ± 0.50^b^	13.69 ± 0.52^b^	47.28 ± 0.19^b^

*Note:* After the value, different letters indicate significant difference (*p* < 0.05).

## 4. Conclusion

The results confirmed that the AMRC developed in this study possessed favorable physicochemical properties. It was nutritionally enhanced, as evidenced by significantly higher levels of ash, protein, and carbohydrates, while concurrently exhibiting a significantly lower fat content compared to both control samples. The AMRC sample exhibited greater hardness and chewiness compared to the two control rice crusts. Color analysis demonstrated that the addition of *Astragalus membranaceus* powder notably increased the yellowness (*b*∗ value) and lightness (*L*∗ value) of the rice crust. No significant differences were detected in salty, umami, or richness attributes across the three rice crust variants. The aroma profile of AMRC was primarily composed of aromatic hydrocarbons, alcohols, and alkanes, which was closely aligned with that of CRC. In contrast, compared to MRC, AMRC showed higher responses in sensors W1S (short‐chain alkanes), W2S (alcohols), W3S (long‐chain alkanes and aliphatics), and W5S (nitrogen oxides). SEM analysis demonstrated that the prepared AMRC exhibited a more porous architecture with significantly reduced oil content. The AMRC was significantly enriched in bioactive compounds and exhibited a potent antioxidant capacity relative to the control samples. The developed AMRC demonstrates strong potential to fulfill market needs. This work not only offers strategic insights for *Astragalus membranaceus*–based product innovation but also paves the way for enhancing the overall development level of the *Astragalus membranaceus* industry.

## Conflicts of Interest

The authors declare no conflicts of interest.

## Funding

The study is supported by the Science and Technology Department of Shanxi Province, a key research and development project of Shanxi Province (202302140601015‐2), and the horizontal science and technology project of Shanxi Agricultural University (2023HX038 and 2024HX076).

## Data Availability

Research data are not shared.

## References

[1] Fu J. , Zhang Y. , Hu Y. , Zhao G. , Tang Y. , and Zou L. , Concise Review: Coarse Cereals Exert Multiple Beneficial Effects on Human Health, Food Chemistry. (2020) 325, 126761, 10.1016/j.foodchem.2020.126761, 32387947. 32387947

[2] Tomić J. , Torbica A. , and Belović M. , Effect of Non-Gluten Proteins and Transglutaminase on Dough Rheological Properties and Quality of Bread Based on Millet (Panicum miliaceum) Flour, LWT. (2020) 118, 108852, 10.1016/j.lwt.2019.108852.

[3] Pawde S. , Talib M. I. , and Parate V. R. , Development of Fiber-Rich Biscuit by Incorporating Dragon Fruit Powder, International Journal of Fruit Science. (2020) 20, no. sup3, S1620–S1628, 10.1080/15538362.2020.1822267.

[4] Hidalgo A. , Brandolini A. , Čanadanović-Brunet J. , Ćetković G. , and Šaponjac V. T. , Microencapsulates and Extracts From Red Beetroot Pomace Modify Antioxidant Capacity, Heat Damage and Colour of Pseudocereals-Enriched Einkorn Water Biscuits, Food Chemistry. (2018) 268, 40–48, 10.1016/j.foodchem.2018.06.062, 2-s2.0-85048751940, 30064775. 30064775

[5] Indrianingsih A. W. , Khasanah Y. , Darsih C. , Hastuti H. P. , Suryani A. E. , Hastuti H. , Ni’maturrohmah D. , Laila U. , Noviana E. , Rahayu E. , and Wiyono E. , Sorghum Cookies Fortified With Garcinia mangostana Peel Extract: Formulation, Characterization, and Evaluation of Antioxidant and Antidiabetic Activity, Bioactive Carbohydratesand Dietary Fibre. (2025) 33, 100467, 10.1016/j.bcdf.2025.100467.

[6] Dong M. , Li J. , Yang D. , Li M. , and Wei J. , Biosynthesis and Pharmacological Activities of Flavonoids, Triterpene Saponins and Polysaccharides Derived From Astragalus membranaceus, Molecules. (2023) 28, no. 13, 10.3390/molecules28135018, 37446680. PMC10343288 37446680

[7] Chen G. , Jiang N. , Zheng J. , Hu H. , Yang H. , Lin A. , Hu B. , and Liu H. , Structural Characterization and Anti-Inflammatory Activity of Polysaccharides From Astragalus membranaceus, International Journal of Biological Macromolecules. (2023) 241, 124386, 10.1016/j.ijbiomac.2023.124386, 37054858. 37054858

[8] Graziani V. , Scognamiglio M. , Esposito A. , Fiorentino A. , and D’Abrosca B. , Chemical Diversity and Biological Activities of the Saponins Isolated From Astragalus Genus: Focus on Astragaloside IV, Phytochemistry Reviews. (2019) 18, no. 4, 1133–1166, 10.1007/s11101-019-09626-y, 2-s2.0-85068860942.

[9] Cong B. , Perspectives in Food & Medicine Homology, Food & Medicine Homology. (2024) 1, no. 1, 9420018, 10.26599/FMH.2024.9420018.

[10] Wei F. , Wang H. , Li X. , Cao J. , and Zhang X. , Preparation of Astragalus membranaceus-Cranberry Biscuits and the Evaluation of Physicochemical Properties and Antioxidant Activity, International Journal of Food Science and Technology. (2024) 59, no. 5, 3134–3141, 10.1111/ijfs.17057.

[11] Sun-Waterhouse D. X. , Chen X. Y. , Liu Z. H. , Waterhouse G. I. , and Kang W. Y. , Transformation From Traditional Medicine-Food Homology to Modern Food-Medicine Homology, Food and Medicine Homology. (2024) 1, no. 1, 9420014, 10.26599/FMH.2024.9420014.

[12] Song B. N. , Park S. K. , Lee S. H. , Park B. R. , Park C. S. , and Park S. Y. , Quality Characteristics and Antioxidant Activity of Germinated Black Rice Porridge Based on the Amount of Astragalus membranaceus (Fisch.) Bunge and Fermented A. membranaceus (Fisch.) Bunge Added, Journal of the Korean Society of Food Science and Nutrition. (2023) 52, no. 12, 1274–1281, 10.3746/jkfn.2023.52.12.1274.

[13] Wang J. , Kong X. , Han Y. , Sam F. E. , Li J. , Qi Z. , and Jiang Y. , Ultrasonic Replacement of Natural Aging: Potential Strategies for Improving the Color, Antioxidant Activity, and Volatile Compound Profile of Astragalus Mead, Ultrasonics Sonochemistry. (2025) 116, 107319, 10.1016/j.ultsonch.2025.107319, 40121708. 40121708 PMC11981771

[14] AOAC International and Latimer G. W. , Official Methods of Analysis of AOAC International, 2023, AOAC International, 10.1093/9780197610145.002.001.

[15] Chang S. K. C. and Zhang Y. , Protein Analysis, Food Analysis, 2017, Springer International Publishing, Cham, 315–331, 10.1007/978-3-319-45776-5_18.

[16] Arepally D. , Reddy R. S. , Coorey R. , and Goswami T. , Evaluation of Functional, Physicochemical, Textural and Sensorial Properties of Multi-Millet-Based Biscuit, International Journal of Food Science and Technology. (2023) 58, no. 5, 2437–2447, 10.1111/ijfs.16381.

[17] Lu Z. , Chen L. , Bai C. , Yuan M. , Jiang Y. , and Zhao L. , Changes of Taste and Characteristic Flavor of Vinasse Fish During Different Distiller′s Grains Time, Journal of Food Composition and Analysis. (2025) 139, 107162, 10.1016/j.jfca.2024.107162.

[18] Zhao Z. , Hao Y. , Liu Y. , Shi Y. , Lin X. , Wang L. , Wen P. , Hu X. , and Li J. , Comprehensive Evaluation of Aroma and Taste Properties of Different Parts From the Wampee Fruit, Food Chemistry: X. (2023) 19, 100835, 10.1016/j.fochx.2023.100835, 37636899. 37636899 PMC10457502

[19] Cui L. , Ma Z. , Wang D. , and Niu Y. , Ultrasound-Assisted Extraction, Optimization, Isolation, and Antioxidant Activity Analysis of Flavonoids From Astragalus membranaceus Stems and Leaves, Ultrasonics Sonochemistry. (2022) 90, 106190, 10.1016/j.ultsonch.2022.106190, 36215890. 36215890 PMC9554832

[20] Ji Y. B. , Ru X. , Yu M. , Wang S. , Lu L. , Qiao A. , and Guo S. , Extraction and Determination of Total Flavonoids in Jujube by Alcohol Extraction, IOP Conference Series: Earth and Environmental Science. (2017) 100, no. 1, 012054, 10.1088/1755-1315/100/1/012054, 2-s2.0-85039450083.

[21] Li W. , Shao C. , Huang P. , Yu D. , Yang J. , Wan H. , and He Y. , Optimization, Characterization of Astragalus Polysaccharides, and Evaluation of Anti-Inflammation Effect in Primary Cultured Astrocytes via HMGB1/RAGE/NF-*κ*B/NLRP3 Signal Pathway, Industrial Crops and Products. (2023) 197, 116594, 10.1016/j.indcrop.2023.116594.

[22] Chen F. and Huang G. , Antioxidant Activity of Polysaccharides From Different Sources of Ginseng, International Journal of Biological Macromolecules. (2019) 125, 906–908, 10.1016/j.ijbiomac.2018.12.134, 2-s2.0-85058970116. 30572039

[23] Hu Y. , Cui X. , Zhang Z. , Chen L. , Zhang Y. , Wang C. , Yang X. , Qu Y. , and Xiog Y. , Optimisation of Ethanol-Reflux Extraction of Saponins From Steamed Panax notoginseng by Response Surface Methodology and Evaluation of Hematopoiesis Effect, Molecules. (2018) 23, no. 5, 10.3390/molecules23051206, 2-s2.0-85047187458, 29772847. PMC6099958 29772847

[24] de Aguiar N. S. , Hanse F. A. , Reis C. A. F. , Lazzarotto M. , and Wendling I. , Optimizing the Vanillin-Acid Sulfuric Method to Total Saponin Content in Leaves of Yerba Mate Clones, Chemistry and Biodiversity. (2024) 21, no. 4, 10.1002/cbdv.202301883, e202301883, 38358959. 38358959

[25] Gramza-Michałowska A. , Kobus-Cisowska J. , Kmiecik D. , Korczak J. , Helak B. , Dziedzic K. , and Górecka D. , Antioxidative Potential, Nutritional Value and Sensory Profiles of Confectionery Fortified With Green and Yellow Tea Leaves (Camellia sinensis), Food Chemistry. (2016) 211, 448–454, 10.1016/j.foodchem.2016.05.048, 2-s2.0-84969217066, 27283654. 27283654

[26] Szydłowska-Czerniak A. , Poliński S. , and Momot M. , Optimization of Ingredients for Biscuits Enriched With Rapeseed Press Cake—Changes in Their Antioxidant and Sensory Properties, Applied Sciences. (2021) 11, no. 4, 10.3390/app11041558.

[27] Re R. , Pellegrini N. , Proteggente A. , Pannala A. , Yang M. , and Rice-Evans C. , Antioxidant Activity Applying an Improved ABTS Radical Cation Decolorization Assay, Free Radical Biology and Medicine. (1999) 26, no. 9-10, 1231–1237, 10.1016/S0891-5849(98)00315-3, 2-s2.0-0032982508, 10381194. 10381194

[28] Ding L. , Zhang X. , and Zhang J. , Antioxidant Activity In Vitro Guided Screening and Identification of Flavonoids Antioxidants in the Extract From Tetrastigma hemsleyanum Diels et Gilg, International Journal of Analytical Chemistry. (2021) 2021, 10.1155/2021/7195125, 7195125, 34858501. 34858501 PMC8632396

[29] Papadaki E. and Roussis I. G. , Assessment of Antioxidant and Scavenging Activities of Various Yogurts Using Different Sample Preparation Procedures, Applied Sciences. (2022) 12, no. 18, 10.3390/app12189283.

[30] Indrianingsih A. W. , Rosyida V. T. , Darsih C. , Apriyana C. , Iwansyah A. C. , Khasanah Y. , Kusumaningrum A. , Windarsih A. , Herawati E. R. N. , Muzdalifah D. , and Sulistyowaty M. I. , Physicochemical Properties, Antioxidant Activities, *β*-Carotene Content, and Sensory Properties of Cookies From Pumpkin (Cucurbita moschata) and Modified Cassava Flour (Manihot esculenta), Bioactive Carbohydrates and Dietary Fibre. (2024) 31, 100398, 10.1016/j.bcdf.2023.100398.

[31] Sharma B. and Gujral H. S. , Modulation in Quality Attributes of Dough and Starch Digestibility of Unleavened Flat Bread on Replacing Wheat Flour With Different Minor Millet Flours, International Journal of Biological Macromolecules. (2019) 141, 117–124, 10.1016/j.ijbiomac.2019.08.252, 2-s2.0-85071657398, 31476390. 31476390

[32] Wang S. , Peng Y. , Zhuang Y. , Wang N. , Jin J. , and Zhan Z. , Purification, Structural Analysis and Cardio-Protective Activity of Polysaccharides From Radix Astragali, Molecules. (2023) 28, no. 10, 10.3390/molecules28104167. PMC10222213 37241906

[33] Sogabe T. , Kobayashi R. , Thanatuksorn P. , Suzuki T. , and Kawai K. , Physical and Structural Characteristics of Starch-Based and Conventional Cookies: Water Sorption, Mechanical Glass Transition, and Texture Properties of Their Crust and Crumb, Journal of Texture Studies. (2021) 52, no. 3, 347–357, 10.1111/jtxs.12585, 33464561. 33464561

[34] Mota J. , Lima A. , Ferreira R. B. , and Raymundo A. , Technological Potential of a Lupin Protein Concentrate as a Nutraceutical Delivery System in Baked Cookies, Foods. (2021) 10, no. 8, 10.3390/foods10081929, 34441706. PMC8393273 34441706

[35] Han S. , Mei H. , Chen S. , Bai Z. , Yue C. , Wang L. , Li P. , and Luo D. , Effect of Particle Size of Eucommia ulmoides Leaf Micro-Powder on Dough Characteristics and Biscuit Quality of Shortbread Biscuits, Food Chemistry: X. (2025) 29, 10.1016/j.fochx.2025.102715, 40686873. PMC12272924 40686873

[36] Sari K. I. and Rafisa A. , Chewing and Swallowing Patterns for Different Food Textures in Healthy Subjects, International Journal of Dentistry. (2023) 2023, 6709350, 10.1155/2023/6709350, 37361412. 37361412 PMC10290560

[37] Nasiri F. , Mohtarami F. , Esmaiili M. , and Pirsa S. , Production of Gluten-Free Biscuits With Inulin and Flaxseed Powder: Investigation of Physicochemical Properties and Formulation Optimization, Biomass Conversion and Biorefinery. (2024) 14, no. 17, 21443–21459, 10.1007/s13399-023-04297-4.

[38] Göksu F. , Özlü Z. , and Bölek S. , Rhubarb Powder: Potential Uses as a Functional Bread Ingredient, Journal of Food Science. (2024) 89, no. 4, 2017–2024, 10.1111/1750-3841.16987, 38488728. 38488728

[39] Kouhsari F. , Saberi F. , Kowalczewski P. Ł. , Lorenzo J. M. , and Kieliszek M. , Effect of the Various Fats on the Structural Characteristics of the Hard Dough Biscuit, Lwt. (2022) 159, 113227, 10.1016/j.lwt.2022.113227.

[40] Manzocco L. , Calligaris S. , Mastrocola D. , Nicoli M. C. , and Lerici C. R. , Review of Non-Enzymatic Browning and Antioxidant Capacity in Processed Foods, Trends in Food Science and Technology. (2000) 11, no. 9-10, 340–346, 10.1016/S0924-2244(01)00014-0, 2-s2.0-0034633084.

[41] Li Y. , Jongberg S. , Andersen M. L. , Davies M. J. , and Lund M. N. , Quinone-Induced Protein Modifications: Kinetic Preference for Reaction of 1, 2-Benzoquinones With Thiol Groups in Proteins, Free Radical Biology and Medicine. (2016) 97, 148–157, 10.1016/j.freeradbiomed.2016.05.019, 2-s2.0-84973304277, 27212016. 27212016

[42] Gong G. , Zheng Y. , Yang Y. , Sui Y. , and Wen Z. , Pharmaceutical Values of Calycosin: One Type of Flavonoid Isolated From Astragalus, Evidence-Based Complementary and Alternative Medicine. (2021) 2021, 9952578, 10.1155/2021/9952578, 34035829. 34035829 PMC8121564

[43] Devi A. and Khatkar B. S. , Physicochemical, Rheological and Functional Properties of Fats and Oils in Relation to Cookie Quality: A Review, Journal of Food Science and Technology. (2016) 53, no. 10, 3633–3641, 10.1007/s13197-016-2355-0, 2-s2.0-84991401939, 28017978. 28017978 PMC5147699

[44] Cai W. , Tang F. , Guo Z. , Guo X. , Zhang Q. , Zhao X. , Ning M. , and Shan C. , Effects of Pretreatment Methods and Leaching Methods on Jujube Wine Quality Detected by Electronic Senses and HS-SPME-GC-MS, Food Chemistry. (2020) 330, 127330, 10.1016/j.foodchem.2020.127330, 32569941. 32569941

[45] Razak M. and Pudjirahaju A. , Effect of Substitution of Cowpea Sprouts Flour (Vigna unguiculata L.) and Sorghum Flour (Sorghum bicolor L.) of Chemical Quality and Organoleptic Quality on Biscuits as Supplementary Feeding Recovery for Protein Energy Malnutrition School Age Children, Journal of Local Therapy. (2024) 3, no. 1, 23–32, 10.31290/jlt.v3i1.4364.

[46] Osakabe N. , Shimizu T. , Fujii Y. , Fushimi T. , and Calabrese V. , Sensory Nutrition and Bitterness and Astringency of Polyphenols, Biomolecules. (2024) 14, no. 2, 10.3390/biom14020234, 38397471. PMC10887135 38397471

[47] Verma R. C. , Jitendrakumar P. H. , Ashoka P. , Shekhar S. , Pal A. , Mondal K. , Panotra N. , and Singh B. V. , A Review of Millet Grain Phenolics, Their Health Promotion and Disease Risk Reduction, International Journal of Plant and Soil Science. (2023) 35, no. 20, 863–874, 10.9734/ijpss/2023/v35i203878.

[48] Tiggemann L. , Ballen S. C. , Bocalon C. M. , Graboski A. M. , Manzoli A. , Steffens J. , Valduga E. , and Steffens C. , Electronic Nose System Based on Polyaniline Films Sensor Array With Different Dopants for Discrimination of Artificial Aromas, Innovative Food Science & Emerging Technologies. (2017) 43, 112–116, 10.1016/j.ifset.2017.08.003, 2-s2.0-85029129559.

[49] Sun H. , Xie D. , Guo X. , Zhang L. , Li Z. , Wu B. , and Qin X. , Study on the Relevance Between Beany Flavor and Main Bioactive Components in Radix Astragali, Journal of Agricultural and Food Chemistry. (2010) 58, no. 9, 5568–5573, 10.1021/jf9042634, 2-s2.0-77952202365, 20359230. 20359230

[50] Hitlamani V. , Hemraj T. M. , and Inamdar A. A. , Optimizing Fatty Acid Composition in Cookie Formulation Using Vegetable Oil Blends: Impacts on Dough Rheology, Physical Properties, and Sensory Qualities, Journal of Food Measurement and Characterization. (2025) 19, no. 4, 2461–2475, 10.1007/s11694-025-03124-w.

[51] Devi A. and Khatkar B. S. , Effects of Fatty Acids Composition and Microstructure Properties of Fats and Oils on Textural Properties of Dough and Cookie Quality, Journal of Food Science and Technology. (2018) 55, no. 1, 321–330, 10.1007/s13197-017-2942-8, 2-s2.0-85032222767, 29358825. 29358825 PMC5756218

[52] Ahmad M. , Baba W. N. , Wani T. A. , Gani A. , Gani A. , Shah U. , Wani S. M. , and Masoodi F. A. , Effect of Green Tea Powder on Thermal, Rheological & Functional Properties of Wheat Flour and Physical, Nutraceutical & Sensory Analysis of Cookies, Journal of Food Science and Technology. (2015) 52, no. 9, 5799–5807, 10.1007/s13197-014-1701-3, 2-s2.0-84940093275, 26344994. 26344994 PMC4554617

[53] Chen H. , Li H. , Chen K. , Wang Z. , Fu M. , and Kan J. , Effect of Oleic Acid-Rich Rapeseed Oil on the Physicochemical, Rheological, and Structural Characteristics of Wheat Dough, Food Chemistry. (2024) 458, 140227, 10.1016/j.foodchem.2024.140227, 38943950. 38943950

[54] Quintelas C. , Rodrigues C. , Sousa C. , Ferreira E. C. , and Amaral A. L. , Cookie Composition Analysis by Fourier Transform Near Infrared Spectroscopy Coupled to Chemometric Analysis, Food Chemistry. (2024) 435, 137607, 10.1016/j.foodchem.2023.137607, 37778254. 37778254

[55] Safar M. , Bertrand D. , Robert P. , Devaux M. F. , and Genot C. , Characterization of Edible Oils, Butters and Margarines by Fourier Transform Infrared Spectroscopy With Attenuated Total Reflectance, Journal of the American Oil Chemists’ Society. (1994) 71, no. 4, 371–377, 10.1007/bf02540516, 2-s2.0-0028407994.

[56] Pradhan A. , Anis A. , Alam M. A. , Al-Zahrani S. M. , Jarzebski M. , and Pal K. , Effect of Soy Wax/Rice Bran Oil Oleogel Replacement on the Properties of Whole Wheat Cookie Dough and Cookies, Foods. (2023) 12, no. 19, 10.3390/foods12193650, 37835303. PMC10572930 37835303

[57] Sevenou O. , Hill S. E. , Farhat I. A. , and Mitchell J. R. , Organisation of the External Region of the Starch Granule as Determined by Infrared Spectroscopy, International Journal of Biological Macromolecules. (2002) 31, no. 1-3, 79–85, 10.1016/S0141-8130(02)00067-3, 2-s2.0-0037147685, 12559430. 12559430

[58] Pulatsu E. , Su J. W. , Kenderes S. M. , Lin J. , Vardhanabhuti B. , and Lin M. , Effects of Ingredients and Pre-Heating on the Printing Quality and Dimensional Stability in 3D Printing of Cookie Dough, Journal of Food Engineering. (2021) 294, 110412, 10.1016/j.jfoodeng.2020.110412.

[59] Möwes M. , Kandanda G. K. , Nangolo L. N. , Shafodino F. S. , and Mwapagha L. M. , Qualitative Phytochemical Profiling, and In Vitro Antimicrobial and Antioxidant Activity of Psidium guajava (Guava), PLoS One. (2025) 20, no. 4, e0321190, 10.1371/journal.pone.0321190, 40193371. 40193371 PMC11975133

[60] Rito M. , Marques J. , da Costa R. M. , Correia S. , Lopes T. , Martin D. , Canhoto J. , Batista de Carvalho L. , and Marques M. , Antioxidant Potential of Tamarillo Fruits—Chemical and Infrared Spectroscopy Analysis, Antioxidants. (2023) 12, no. 2, 10.3390/antiox12020536, 36830094. PMC9952541 36830094

[61] Gai Q. , Feng X. , Jiao J. , Xu X. , Fu J. , He X. , and Fu Y. , Blue LED Light Promoting the Growth, Accumulation of High-Value Isoflavonoids and Astragalosides, Antioxidant Response, and Biosynthesis Gene Expression in Astragalus membranaceus (Fisch.) Bunge Hairy Root Cultures, Plant Cell, Tissue and Organ Culture (PCTOC). (2023) 153, no. 3, 511–523, 10.1007/s11240-023-02486-7, 37197002. 37197002 PMC10042671

[62] Sharma R. , Sharma S. , Dar B. N. , and Singh B. , Millets as Potential Nutri-Cereals: A Review of Nutrient Composition, Phytochemical Profile and Techno-Functionality, International Journal of Food Science and Technology. (2021) 56, no. 8, 3703–3718, 10.1111/ijfs.15044.

[63] Yashin A. , Yashin Y. , Xia X. , and Nemzer B. , Antioxidant Activity of Spices and Their Impact on Human Health: A Review, Antioxidants. (2017) 6, no. 3, 10.3390/antiox6030070, 2-s2.0-85029738237, 28914764. PMC5618098 28914764

[64] Pan S. , Gao R. , and Wu S. , Preparation, Characterization and Hypolipidaemic Activity of Astragalus membranaceus Polysaccharide, Journal of Functional Foods. (2017) 39, 264–267, 10.1016/j.jff.2017.10.033, 2-s2.0-85032271667.

[65] Nafti K. , Giacinti G. , Marghali S. , and Raynaud C. D. , Screening for Astragalus hamosus Triterpenoid Saponins Using HPTLC Methods: Prior Identification of Azukisaponin Isomers, Molecules. (2022) 27, no. 17, 10.3390/molecules27175376, 36080144. PMC9457977 36080144

[66] Kaur H. , Oberoi H. K. , Ganapathy K. N. , and Bhardwaj R. , Effect of Popping and Malting Processing Techniques on Physiochemical, Antinutrients and Antioxidant Properties of Millets Flour, Journal of Food Science and Technology. (2023) 60, no. 9, 2370–2384, 10.1007/s13197-023-05758-4, 37424574. 37424574 PMC10326190

[67] Zielińska E. and Pankiewicz U. , Nutritional, Physiochemical, and Antioxidative Characteristics of Shortcake Biscuits Enriched With Tenebrio Molitor Flour, Molecules. (2020) 25, no. 23, 10.3390/molecules25235629. PMC7730627 33265946

[68] Sheng Z. , Jiang Y. , Liu J. , and Yang B. , UHPLC-MS/MS Analysis on Flavonoids Composition in Astragalus membranaceus and Their Antioxidant Activity, Antioxidants. (2021) 10, no. 11, 10.3390/antiox10111852, 34829723. PMC8614773 34829723

[69] Yao J. , Peng T. , Shao C. , Liu Y. , Lin H. , and Liu Y. , The Antioxidant Action of Astragali Radix: Its Active Components and Molecular Basis, Molecules. (2024) 29, no. 8, 10.3390/molecules29081691, 38675511. PMC11052376 38675511

[70] Samuel A. O. , Huang B. T. , Chen Y. , Guo F. , Yang D. , and Jin J. , Antioxidant and Antibacterial Insights Into the Leaves, Leaf Tea and Medicinal Roots From Astragalus membranaceus (Fisch.) Bge, Scientific Reports. (2021) 11, no. 1, 19625, 10.1038/s41598-021-97109-6, 34608170. 34608170 PMC8490359

